# Three-Dimensional Echocardiographic Assessment of Right Ventricular Global Myocardial Work and Ventricular–Pulmonary Coupling in ATTR Cardiac Amyloidosis

**DOI:** 10.3390/jcm14030668

**Published:** 2025-01-21

**Authors:** Ashwin Venkateshvaran, Fredrik Edbom, Sandra Arvidsson, Attila Kovacs, Per Lindqvist

**Affiliations:** 1 Clinical Physiology, Department of Clinical Sciences Lund, Lund University, Skåne University Hospital, 22185 Lund, Sweden; 2 Department of Diagnostics and Intervention, Clinical Physiology, Umeå University, 90187 Umeå, Swedenper.lindqvist@umu.se (P.L.); 3 Argus Cognitive, Inc., Hanover, NH 03755, USA; 4 Department of Experimental Cardiology and Surgical Techniques, Semmelweis University, 1085 Budapest, Hungary

**Keywords:** cardiac amyloidosis, right ventricular function, echocardiography, ventricular–arterial coupling

## Abstract

**Background:** Right ventricular (RV) function is inadequately investigated and routinely overlooked in transthyretin amyloid cardiomyopathy (ATTR-CM). Novel imaging distinguishers between intrinsic RV myocardial disease in ATTR-CM and primary RV overload disorder phenotypes may enhance mechanistic and pathophysiological understanding of RV dysfunction. We aimed to investigate RV performance in ATTR-CM employing comprehensive 2D and 3D echocardiography, and to compare these indices with primary RV afterload disease. **Methods:** We investigated conventional and novel indices of RV contractile function, myocardial work and ventricular–vascular coupling in 21 well-characterized ATTR-CM patients, 10 PAH patients and 12 healthy controls. RV long axis function and pulmonary artery (PA) systolic pressure were evaluated using 2D Doppler echocardiography. RV ejection fraction (RVEF), volumes, global longitudinal strain (GLS) and novel myocardial work indices were analyzed by 3D echocardiography. RV elastance (E_es_), afterload (E_a_) and RV-PA coupling (E_es_/E_a_) were estimated using the single-beat volume method. **Results:** ATTR-CM showed lower RVEF, GLS and E_es_, and a higher RV global myocardial work index (GWI), constructive work (GCW), E_a_ and reduced RV-PA coupling compared with controls. RV EF, stroke volume, GLS and circumferential strain did not differ between ATTR-CM and PAH. However, GWI, GCW, E_es_ and E_a_ were lower in ATTR-CM. RV–pulmonary coupling displayed strong association with RV 3D strain (r = 0.84, *p* < 0.001), whereas RV E_es_ (contractility) was related to RV GWI (r = 0.54, *p* < 0.001). **Conclusions:** ATTR-CM displayed lower RV performance, higher GMW and reduced RV-PA coupling. Myocardial work indices E_es_ and E_a_ are novel distinguishers of RV dysfunction phenotypes. The clinical and prognostic value of these novel variables warrant further investigation.

## 1. Background

Evaluation of right ventricular (RV) function plays a critical role in disease management and prognosis in heart failure (HF). Deteriorating RV function is associated with poor exercise tolerance [1] and is an independent predictor of adverse outcome [2], HF-related hospitalization [3] and sudden cardiac death [4].

In recent years, altered RV function owing to amyloid infiltration has been shown to impact clinical prognosis and hospitalization rates in transthyretin cardiac amyloidosis (ATTR-CM) [5,6]. However, comprehensive characterization of RV function in ATTR-CM is relatively unexplored. Further, RV myocardial work and right-heart ventricular–vascular interaction have emerged as more robust representations of RV function, as they account for loading conditions that impact conventional RV performance metrics during routine assessment [7]. Studies that evaluated load-independent measures in cardiac amyloidosis have limited their investigation to the left heart [8,9,10]. To our knowledge, no studies provide information on RV myocardial work and right-heart ventricular–vascular interaction in cardiac amyloidosis.

Alterations to RV structure and function are multifactorial and may be secondary to the intrinsic myocardial disease seen in infiltrative cardiomyopathy or owing to elevated RV afterload observed in pulmonary arterial hypertension (PAH). Irrespective of whether RV dysfunction is secondary to myocardial or pulmonary disease, it degenerates rapidly when left untreated. Novel imaging distinguishers between intrinsic RV myocardial disease and load-impacted RV function may offer mechanistic and pathophysiological insight that can potentially contribute to tailored therapy and optimize disease management.

Advances in 3D echocardiography permit detailed characterization of RV volume, ejection fraction (EF) and RV global longitudinal strain. Further, offline analysis based on 2D and 3D data permits evaluation of myocardial work indices, RV elastance and RV-pulmonary artery (PA) coupling. Our primary objective was to examine the extent of alterations in RV myocardial work and RV–pulmonary coupling in ATTR-CM. Additionally, we aimed to compare indices of RV performance between two models: one representing RV infiltrative disease combined with the overload phenomena characteristic of ATTR-CM, and the other predominantly reflecting RV afterload disease as seen in PAH.

## 2. Methods

Thirty cardiac amyloidosis patients were retrospectively selected from ATTR patient clinics undergoing evaluation at Umeå University Hospital between 2018 and 2024. All ATTR-CM patients had undergone a transthoracic echocardiographic examination which demonstrated an interventricular septal thickness (IVST) >14 mm. All ATTR-CM patients had a DPD scintigraphy-verified diagnosis. Hematologic testing ruled out AL amyloidosis. Genetic testing identified 16 of 21 patients as having hereditary ATTR (ATTRv) and all had the genetic mutation Val30Met. In addition, 20 patients with PH, defined as resting mean pulmonary artery pressure of more than 20 mmHg estimated on right-heart catheterization, were included. Further, 15 healthy controls were included for comparison. The control group comprised of a subset of individuals originally recruited to the Umeå General Population Heart Study [11]. None of the controls had any cardiovascular or systemic disease and did not use any medications known to influence cardiac function. Patients with technically inadequate images for 3D capture and evaluation were subsequently excluded from the analysis.

The study complied with the Declaration of Helsinki and was approved by the Regional Ethical Committee at Umeå University, Sweden (Dnr 07−092 M, 2 October 2007). Written informed consent was obtained from all study participants.

Echocardiography. All patients and controls underwent a comprehensive echocardiographic examination, including 2D Doppler and 3D echocardiography by a single, experienced operator (P.L.) using a GE Vivid E9 (GE Vingmed Ultrasound, Horten, Norway), while lying in the left lateral decubitus position. Echocardiographic analysis was performed offline using the commercially available software packages Echopac PC version 116 (GE Healthcare, Horten, Norway) and TomTec Imaging Systems, Version 4.5, (Unterschleissheim, Germany). Data analysis was performed according to the recommendations of the American Society of Echocardiography [12]. From the parasternal long axis view (PLAX), left ventricular (LV) diameters, interventricular septal thickness in diastole (IVST) and posterior wall thickness in diastole (PWT) were measured. From the apical four-chamber view and two-chamber view, LV ventricular ejection fraction (LVEF) was calculated using Simpson’s biplane formula.

Right ventricular (RV) systolic long axis function was assessed by M-mode measured tricuspid annular plane systolic excursion (TAPSE) from the RV free wall. TAPSE was defined as the total longitudinal displacement between the time of the Q-wave of the ECG and end systole (end of T-wave on ECG); TAPSE < 17 mm was a marker of impaired systolic RV longitudinal function. RV systolic pressure was estimated from peak systolic tricuspid regurgitation. RAP was estimated as 7 mmHg in all patients [13].

**Figure 1:**
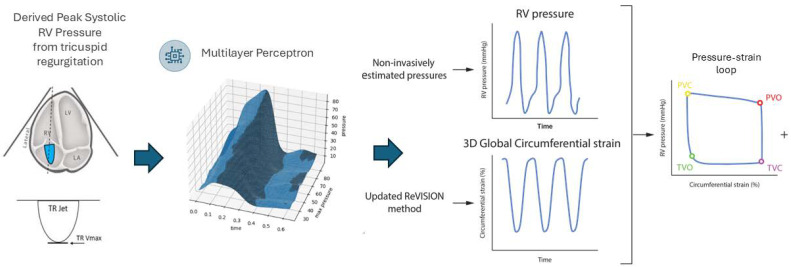
Non-invasive generation of right ventricular (RV) pressure–strain loop. The image to the far left displays the capture of a tricuspid regurgitation signal using Continuous Wave Doppler. RV = right ventricle, PVO = pulmonary valve opening, PVC = pulmonary valve closure, TV = tricuspid valve opening, TVC = tricuspid valve closure.

Three-dimensional echocardiography datasets were analyzed using the 4D RV-Function 2 software (TomTec Imaging GmbH, Unterschleissheim, Germany) to calculate RV volumes and EF in keeping with current recommendations [14]. The reconstructed 3D mesh models were imported into the ReVISION software https://revi.cc/ (Argus Cognitive, Inc., Hanover, NH, USA), employing a previously reported and validated methodology [15,16]. Using the ReVISION software, we assessed 3D-based RV global circumferential and longitudinal strain and myocardial work indices. To create a pressure–strain loop and calculate related work indices, we needed a pressure curve for the entire cardiac cycle to concatenate with the strain curve. A non-invasive RV pressure curve was reconstructed using the peak systolic RV pressure value as previously described [17]. Briefly, the peak systolic RV pressure was used as the single input for a validated multilayer perceptron model that reconstructed the pressure curve for the entire cardiac cycle along with valvular events. This approach has been validated against gold-standard pressure–volume analysis in earlier studies employing invasive pressure-conductance measurements of the right heart [18]. The pressure–longitudinal strain relationship-derived myocardial work was quantified as the area under the curve and characterized by the following metrics: global work index (GWI), global constructive work (GCW) and global wasted work (GCW). An illustration of the methodology is provided in Figure 1.

RV elastance (E_es_) was estimated as the ratio between RV end-systolic right ventricular pressure (sPAPx0.9) obtained by 2D Doppler echocardiography from RV-RA pressure drop and RV stroke volume (RVSV) from RV 3D adopting the previously validated single-beat method [19]. Arterial elastance (E_a_) was estimated using the ratio between RV end-systolic pressure and RV end systolic volume (RVESV). The RV E_es_/E_a_ ratio was then calculated to represent RV-PA coupling based on the ‘volume method’. Briefly, when RV contractile function cannot increase (reduction in E_es_) to match a high RV afterload (elevated E_a_), RV-PA uncoupling occurs and the E_es_/E_a_ ratio decreases. A value between 1.5 and 2 is considered to represent normal RV-PA coupling. RV-PA coupling was also estimated using the ratio between TAPSE/sPAP as previously published [20].

Statistics. All data were presented as medians and interquartile range (25–75 percentiles), due to the relatively small number of patients investigated. Differences between patient groups were assessed using the Mann–Whitney U test. Correlations between indices for RV-PA coupling, myocardial work and contractility variables were assessed using Spearman’s Rank Correlation. A *p* value < 0.05 was considered statistically significant. Statistical analysis was performed using commercially available software (IBM SPSS statistics, version 22).

## 3. Results

Of 30 cardiac amyloidosis patients, 21 (70%) were technically suitable for performing 3D RV acquisition and thus included in the analysis. Of the 20 PH patients, 9 (45%) displayed optimal images for accurate 3D volume analysis. Among the PH patients, 4 had idiopathic PH and 5 had associated PH. Of the 15 healthy controls, 12 (80%) displayed images suitable for 3D analysis.

### 3.1. ATTR-CM vs. Healthy Controls

Clinical and echocardiographic characteristics of the study population are presented in Table 1. Patients with ATTR-CM were older and displayed higher heart rates than controls. On 2D echocardiography, they had higher LV wall thickness and pulmonary artery pressures, and lower LVEF than controls. Right ventricular function was depressed in the ATTR-CM group, as represented by lower TAPSE and TAPSE/sPAP (*p* < 0.001 for both comparisons).

**Figure 2:**
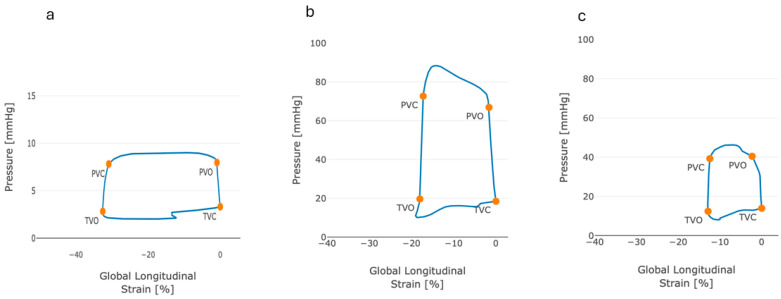
Illustration of right ventricular pressure–strain loops in (**a**) healthy controls, (**b**) PAH patients and (**c**) ATTR-CM patients.

On 3D echocardiography, RV end diastolic volume was higher, and RVEF and RV global longitudinal strain were lower in the ATTR-CM group. Myocardial work indices, represented by the RV global myocardial work index (GWI) and RV global myocardial constructive work (GCW) were higher in the ATTR-CM group compared with controls (*p* < 0.05). RV E_es_ was lower, E_e_ was higher and E_es_/E_a_ was lower in the ATTR-CM group when compared with controls (*p* < 0.05 for all comparisons). Figure 2 displays pressure–strain loops in the ATTR-CM, healthy control and PAH groups.

### 3.2. ATTR-CM vs. PAH

ATTR-CM patients were taller, heavier and displayed higher levels of cardiac biomarkers than PAH patients (*p* < 0.001). On 2D echocardiography, the ATTR-CM group had a thicker LV wall and lower pulmonary artery pressures. TAPSE was lower in the ATTR-CM group compared with the PAH group (*p* = 0.009), but TAPSE/sPAP was similar between patient groups.

On 3D echocardiography, RV end diastolic volume, RVEF, RV stroke volume, RV global and circumferential and longitudinal strain did not differ between ATTR-CM and PAH patients. However, RV GWI and RV GCW were both lower in ATTR-CM patients (*p* < 0.05). Further, both E_a_ and E_es_ were lower in the ATTR-CM group. RV E_es_/E_a_ did not differ between patient groups. Forty-three percent of ATTR-CM and 50% of PAH patients displayed E_es_/E_a_ < 1.

### 3.3. Association Between RV-PA Coupling and RV Performance Markers

**Figure 3:**
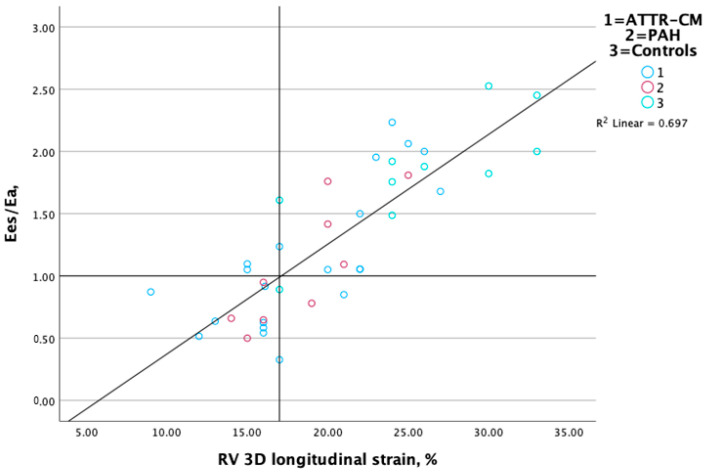
Scatterplot displaying relationship between RV-PA coupling and 3D RV longitudinal strain.

**Figure 4:**
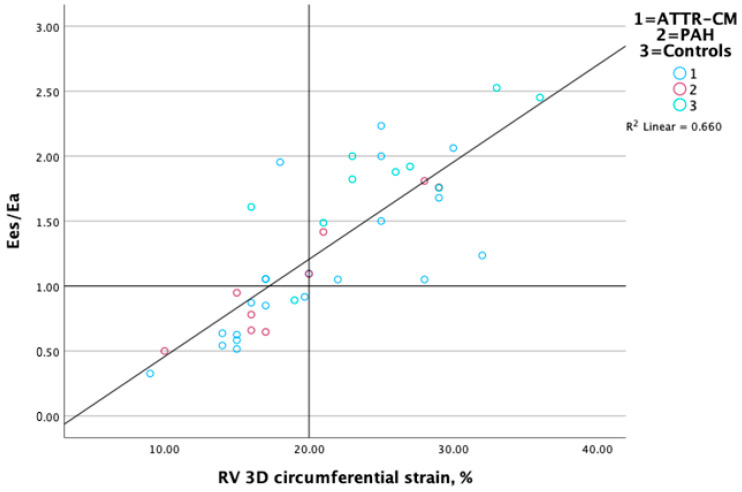
Scatterplot displaying relationship between RV-PA coupling and 3D RV circumferential strain.

RV E_es_/E_a_ (coupling) displayed a strong correlation with RV 3D longitudinal (Figure 3) and circumferential strain (Figure 4) (r = 0.86 and 0.82, respectively, *p* < 0.01) and a more modest correlation with TAPSE (r = 0.73, *p* < 0.05) and TAPSE/PASP (0.61, *p* < 0.05). RV E_es_ measured non-invasively with 3D echocardiography displayed a modest correlation with GWI (r = 0.51, *p* < 0.001). Associations between RV contractile function, ventricular–arterial coupling and RV performance measures are presented in Table 2.

## 4. Discussion

To our knowledge, this is the first study to employ 3D echocardiography to provide a detailed characterization of RV performance, myocardial work and ventricular–pulmonary interaction in ATTR-CM. Our results suggest that ATTR-CM is characterized by reduced RV elastance, elevated arterial afterload, elevated myocardial work indices and RV-PA uncoupling. Impairments in RV EF, volumetric indices and RV-PA coupling in ATTR-CM were similar in both ATTR-CM and PAH. However, E_es_, E_a_ and myocardial work indices were lower in ATTR-CM, suggesting a role for these novel measures to distinguish primary infiltrative RV myocardial disease seen in ATTR-CM from RV impairment due to elevated afterload in PAH. Despite small patient numbers, our findings are hypothesis-generating and provide direction for future physiological and mechanistic studies investigating RV low-performance phenotypes.

Current diagnostic methods for ATTR-cardiac amyloidosis predominantly focus on assessing LV structure and function, often highlighting LV hypertrophy, granular sparkling of the myocardium, diastolic dysfunction, reduced longitudinal strain and regional sparing patterns as hallmark features [21]. Investigators such as de Gregorio et al. suggest that myocardial work analysis offers value in distinguishing hypertrophic phenotypes in the setting of preserved pump performance [22]. However, an approach focused on the LV may overlook the critical role of RV involvement, which can significantly impact prognosis and clinical outcomes in affected patients.

While RV function is often overlooked in cardiac amyloidosis, recent studies suggest a role for RV performance measures in early and differential diagnosis. In AL-amyloidosis, certain studies demonstrate lowered RV longitudinal deformation in patients with cardiac involvement [23] and suggest a role for 3D speckle tracking echocardiography and RV deformation parameters to distinguish AL-amyloidosis from other forms of hypertrophied ventricles [24]. Certain studies suggest that disruption of RV performance may occur simultaneously or even present earlier than the LV in cardiac amyloidosis [25,26,27]. In ATTR-CM, our group demonstrated that segmental RV strain and apex-to-base gradient distinguish ATTR-CM from hypertrophic cardiomyopathy, suggesting a role for it in differential diagnosis [28]. Our current study introduces multiple RV performance variables that are less sensitive to loading and, hypothetically, may outperform traditional RV measures for early and differential diagnosis. This hypothesis needs to be tested in future studies including larger cohorts. Conventional echocardiographic assessment of RV function presents several limitations related to the 2D cut-plane approach, which fails to include all regions of the RV structure. Further, the RV is more susceptible to afterload than the LV, making adaptation of load-independent measures of potential value. Introduction of constructive work, which contributes to RV ejection, wasted work, which encompasses contractions occurring after pulmonic valve closure, and work efficiency as a measure of effective performance provides valuable insights. While these measures are less utilized in clinical practice, their value in diagnosis as presented in our study, and potentially prognosis, highlights the importance of further investigation.

Recent studies in AL-amyloidosis suggest that deteriorating RV involvement displays prognostic significance irrespective of pulmonary hypertension and may support early diagnosis [29,30,31]. Further, a recent position paper from the European Society of Cardiology (ESC) promotes TAPSE as a potential imaging marker for early diagnosis and predicting outcomes in cardiac amyloidosis [32]. In our study, TAPSE displayed a modest correlation with RV-PA coupling when compared with 3D echocardiography-derived strain variables, warranting further exploration of these novel performance measures for stronger diagnostic and prognostic value. Regarding indices of RV contractility, RV E_es_ (ESP/SV) demonstrated a correlation with RV GMWI, suggesting that 3D strain primarily reflects RV–pulmonary coupling, whereas RV GMWI more directly represents RV contractility. This relationship was previously validated in a study validating these measures with invasive pressure–volume assessment [18].

In addition to outcome prediction, RV contractile function measures also predict functional capacity and quality of life. In a recent multivariate analysis in ATTR-CM patients with comprehensive cardiovascular and cardiopulmonary assessment, TAPSE and tissue Doppler-based S-wave velocity were shown to display a strong association with reduced functional capacity during exercise [33]. Given that RV-PA coupling represents the balance between RV mechanical work and oxygen consumption, one can speculate that these measures may display a strong association with cardiopulmonary functional assessment.

In keeping with earlier studies, we show that ATTR-CM patients show RV enlargement, increased pulmonary pressures and lower TAPSE compared with controls [27]. While one can argue that ATTR-CM and PAH have distinct diagnostic and clinical management pathways, they represent physiological phenotypes that both lead to reduced RV performance. In ATTR-CM, RV myocardial infiltration in addition to increased LV filling pressure secondary to LV infiltration induces RV chronic overload. However, PAH can also co-occur in certain cases with amyloid deposition in the pulmonary vasculature [34]. Given the overlap between physiological states that contribute to RV burden, novel imaging distinguishers may contribute to stronger physiological and mechanistic insight. While our study is primarily a physiological study focused on the potential distinction of these RV overload phenotypes, our findings may also have broader implications in identifying ATTR-CM patients with a disproportionately elevated impact on the pulmonary vasculature. This hypothesis will need to be explored in future studies.

Our findings suggest certain clinical implications for the evaluation and management of RV dysfunction in ATTR-CM. First, the use of novel indices, such as RV myocardial work, E_es_, E_a_ and RV–pulmonary artery coupling provides a more nuanced understanding of RV performance beyond conventional measures like ejection fraction and global longitudinal strain. By integrating these advanced echocardiographic measures into clinical practice, clinicians may improve risk stratification, monitor disease progression, and potentially guide therapeutic interventions in ATTR-CM. Additionally, the observed associations between myocardial work indices and RV–pulmonary coupling could offer potential mechanistic insights into RV dysfunction in ATTR-CM. Finally, the potential prognostic value of these novel, load-independent RV performance measures need to be explored in broader clinical settings.

There are several limitations to this study. The small patient population and retrospective study design may limit the generalizability of our findings. Additionally, no tests for inter- or intra-observer variability were possible, given that measurements were based on a single acquisition imported into dedicated software to generate identical results. However, multiple earlier studies have established excellent reproducibility in evaluating RV volumes and function using 3D echocardiography, which formed the basis for this evaluation. The modest feasibility of 3D echocardiography for assessing RV performance indices in PAH remains a potential limitation, as image quality and acquisition challenges may influence measurements. General limitations of myocardial work include its inability to account for wall stress, wall thickness and wall curvature, factors critical to accurately assessing myocardial workload. These limitations are particularly relevant for the RV, whose irregular and complex geometry presents a challenge for precise measurements. Compared to the LV, the RV’s unique structure may result in greater variability and reduced accuracy in myocardial work estimation. Additionally, reliance on imaging-derived pressure and volume parameters, which are more challenging to obtain for the RV, further impacts the precision of RV myocardial work assessment. Finally, our findings are hypothesis-generating and require validation in larger, prospective cohorts with broader representation to confirm their clinical applicability and relevance.

## 5. Conclusions

ATTR-CM displayed lower RV performance, and higher global myocardial work and reduced RV-PA coupling than controls. The myocardial work indices E_es_ and E_a_ are novel distinguishers of RV dysfunction phenotypes. The clinical and prognostic value of these novel variables warrant further investigation.

## Figures and Tables

**Table 1:** Clinical characteristics and echocardiographic data for study population.

	Healthy Controls (n = 12)	Pulmonary Hypertension (n = 9)	ATTR-CM (n = 21)	*p* Value ATTR-CM vs. Controls	*p* Value ATTR-CM vs. PH
Age, years	56 (51–66)	64 (45–74)	69 (63–73)	0.001	ns
Weight, kg	63 (60–78)	77 (62–78)	78 (69–84)	0.051	0.044
Height, cm	172 (167–183)	164 (160–171)	176 (169–183)	ns	0.004
SBP, mmHg	-	125 (116–140)	130 (113–1148)	-	ns
DBP, mmHg	-	75 (69–80)	80 (65–81)	-	ns
HR, bpm	61 (54–69)	74 (67–80)	73 (60–80)	0.045	ns
NT-proBNP, pg/mL	-	256 (61–479)	1418 (438–3372)	-	0.001
Troponin, ng/mL	-	9 (5–29)	39 (29–56)	-	0.004
**2D/Doppler echocardiography**					
Stroke volume, mL	76 (70–80)	75 (61–97)	63 (55–87)	ns	ns
Cardiac output, L/min	4.5 (4.2–5.0)	6.0 (4.5–7.5)	5.1 (3.6–6.7)	ns	ns
LVDd, mm	45 (42–52)	47 (42–50)	41 (39–50)	ns	ns
IVSTd, mm	9 (8–10)	10 (9–11)	20 (16–23)	<0.001	<0.001
PWTd, mm	7 (7–7)	8 (8–9)	14 (12–16)	<0.001	<0.001
LVEF, %	55 (50–60)	55 (53–55)	50 (45–55)	0.027	ns
sPAP, mmHg	25 (20–28)	47 (37–77)	32 (28–40)	<0.001	0.002
TAPSE, mm	24 (20–27)	21 (19–23)	17 (12–20)	<0.001	0.018
TAPSE/sPAP, mm/mmHg	0.96 (0.78–1.22)	0.49 (0.27–0.61)	0.48 (0.35–0.66)	<0.001	ns
**3D RV variables**					
RVDV, mL	100 (76–124)	90 (69–149)	131 (109–143)	0.022	ns
RVEF,%	64 (57–67)	49 (39–60)	50 (38–62)	0.022	ns
RV stroke volume, mL	60 (48–78)	47 (35–67)	60 (48–38)	ns	ns
RV strain circ., %	25 (20–29)	17 (16–25)	18 (15–26)	0.053	ns
RV strain long., %	24 (18–30)	19 (15–20)	17 (15–22)	0.017	ns
GWI, mmHg/%	280 (199–377)	554 (504–973)	446 (336–524)	0.015	0.022
GCW, mmHg/%	270 (210–346)	562 (504–955)	448 (312–518	0.017	0.019
GWW, mmHg/%	11 (6–14)	24 (8–38)	18 (6–32)	ns	ns
RV E_a_, mmHg/mL	0.4 (0.4–0.4)	0.9 (0.6–1.5)	0.5 (0.4–0.8)	0.04	0.006
RV E_es_, mmHg/mL	0.7 (0.5–0.8)	1.1 (0.6–1.6)	0.5 (0.4–0.6)	0.04	0.005
RV E_es_/E_a_	1.8 (1.6–2.1)	0.9 (0.7–1.6)	1.1 (0.6–1.8)	0.005	ns

Data presented as median (Q1–Q3). SBP = systolic blood pressure, DBP = diastolic blood pressure, HR = heart rate, LVDD = Left ventricular diastolic diameter, IVST = interventricular septal thickness, PWTd = posterior wall thickness in diastole, LVEF = left ventricular ejection fraction, sPAP = systolic pulmonary artery pressure, TAPSE = tricuspid annular plane systolic excursion, RVDV = right ventricular end diastolic volume, GWI = RV global myocardial work index, GCW = RV global myocardial constructive work, GWW = global myocardial wasted work, E_a_ = arterial elastance, E_es_ = ventricular elastance.

**Table 2:** Associations between RV contractile function, ventricular–arterial coupling and RV performance measures.

	RV E_es_	RV E_es_/E_a_	TAPSE/sPAP (Coupling)	RV GMWI
RVEF, %	R = 0.41, *p* = 0.08	R = 0.94, *p* < 0.001	R = 0.61, *p* < 0.001	R = −0.14, *p* = 0.36
RV strain C, %	R = 0.26, *p* = 0.10	R = 0.83, *p* > 0.001	R = 0.54, *p* < 0.001	R = −0.16, *p* = 0.32
RV strain L, %	R = 0.24, *p* = 0.13	R = 0.84, *p* < 0.001	R = 0.53, *p* < 0.001	R = −0.08, *p* = 0.62
TAPSE, mm	R = −0.36, *p* = 0.33	R = 0.73, *p* = 0.02	-	R = −0.61, *p* = 0.07
RV GWI, mmHg/%	R = 0.27, *p* = 0.09	R = −0.16, *p* = 0.32	R = 0.51, *p* < 0.001	-

RV = right ventricle, E_es_ = RV elastance, E_a_ = RV afterload, TAPSE = tricuspid annular plane systolic excursion, GWI = RV global myocardial work index.

## Data Availability

The datasets used and/or analyzed during the current study are available from the corresponding author upon reasonable request.

## Abbreviations

ATTR-CM	Transthyretin amyloid cardiomyopathy
E_a_	RV afterload
E_es_	RV elastance
E_es_/E_a_	RV–PA coupling
EF	Ejection fraction
GCW	RV constructive work
GLS	RV global longitudinal strain
GWI	RV global myocardial work index
HF	Heart failure
IVST	Interventricular septal thickness in diastole
PA	Pulmonary artery
PAH	Pulmonary arterial hypertension
PWT	Posterior wall thickness in diastole
RA	Right atrial
RV	Right ventricular
TAPSE	Tricuspid annular plane systolic excursion

## References

[1] Kim J., Di Franco A., Seoane T., Srinivasan A., Kampaktsis P.N., Geevarghese A., Goldburg S.R., Khan S.A., Szulc M., Ratcliffe M.B. (2016). Right ventricular dysfunction impairs effort tolerance independent of left ventricular function among patients undergoing exercise stress myocardial perfusion imaging. Circ. Cardiovasc. Imaging.

[2] Field M.E., Solomon S.D., Lewis E.F., Kramer D.B., Baughman K.L., Stevenson L.W., Tedrow U.B. (2006). Right ventricular dysfunction and adverse outcome in patients with advanced heart failure. J. Card. Fail..

[3] Meluzin J., Spinarová L., Hude P., Krejcí J., Kincl V., Panovský R., Dusek L. (2005). Prognostic importance of various echocardiographic right ventricular functional parameters in patients with symptomatic heart failure. J. Am. Soc. Echocardiogr..

[4] Lella L.K., Sales V.L., Goldsmith Y., Chan J., Iskandir M., Gulkarov I., Tortolani A., Brener S.J., Sacchi T.J., Heitner J.F. (2015). Reduced right ventricular function predicts long-term cardiac re-hospitalization after cardiac surgery. PLoS ONE.

[5] Usuku H., Takashio S., Yamamoto E., Yamada T., Egashira K., Morioka M., Nishi M., Komorita T., Oike F., Tabata N. (2022). Prognostic value of right ventricular global longitudinal strain in transthyretin amyloid cardiomyopathy. J. Cardiol..

[6] Fine N.M., White J.A., Jimenez-Zepeda V., Howlett J.G. (2020). Determinants and prognostic significance of serial right heart function changes in patients with cardiac amyloidosis. Can. J. Cardiol..

[7] He Q., Lin Y., Zhu Y., Gao L., Ji M., Zhang L., Xie M., Li Y. (2023). Clinical usefulness of right ventricle–pulmonary artery coupling in cardiovascular disease. J. Clin. Med..

[8] Bhuiyan T., Helmke S., Patel A.R., Ruberg F.L., Packman J., Cheung K., Grogan D., Maurer M.S. (2011). Pressure-volume relationships in patients with transthyretin (ATTR) cardiac amyloidosis secondary to V122I mutations and wild-type transthyretin: Transthyretin Cardiac Amyloid Study (TRACS). Circ. Heart Fail..

[9] Clemmensen T.S., Eiskjær H., Ladefoged B., Mikkelsen F., Sørensen J., Granstam S.-O., Rosengren S., Flachskampf F.A., Poulsen S.H. (2021). Prognostic implications of left ventricular myocardial work indices in cardiac amyloidosis. Eur. Heart J.-Cardiovasc. Imaging.

[10] Giblin G.T., Cuddy S.A., González-López E., Sewell A., Murphy A., Dorbala S., Falk R.H. (2022). Effect of tafamidis on global longitudinal strain and myocardial work in transthyretin cardiac amyloidosis. Eur. Heart J.-Cardiovasc. Imaging.

[11] Lindqvist P., Waldenström A., Henein M., Mörner S., Kazzam E. (2005). Regional and global right ventricular function in healthy individuals aged 20–90 years: A pulsed Doppler Tissue Imaging Study Umeå General Population Heart Study. Echocardiogr. J. Cardiovasc. Ultrasound Allied Tech..

[12] Lang R.M., Badano L.P., Mor-Avi V., Afilalo J., Armstrong A., Ernande L., Flachskampf F.A., Foster E., Goldstein S.A., Kuznetsova T. (2015). Recommendations for cardiac chamber quantification by echocardiography in adults: An update from the American Society of Echocardiography and the European Association of Cardiovascular Imaging. Eur. Heart J.-Cardiovasc. Imaging.

[13] Venkateshvaran A., Seidova N., Tureli H.O., Kjellström B., Lund L.H., Tossavainen E., Lindquist P. (2021). Accuracy of echocardiographic estimates of pulmonary artery pressures in pulmonary hypertension: Insights from the KARUM hemodynamic database. Int. J. Cardiovasc. Imaging.

[14] Lang R.M., Badano L.P., Tsang W., Adams D.H., Agricola E., Buck T., Faletra F.F., Franke A., Hung J., de Isla L.P. (2012). EAE/ASE recommendations for image acquisition and display using three-dimensional echocardiography. Eur. Heart J.-Cardiovasc. Imaging.

[15] Surkova E., Kovács A., Tokodi M., Lakatos B.K., Merkely B., Muraru D., Ruocco A., Parati G., Badano L.P. (2021). Contraction patterns of the right ventricle associated with different degrees of left ventricular systolic dysfunction. Circ. Cardiovasc. Imaging.

[16] Tokodi M., Staub L., Budai Á., Lakatos B.K., Csákvári M., Suhai F.I., Szabó L., Fábián A., Vágó H., Tősér Z. (2021). Partitioning the right ventricle into 15 segments and decomposing its motion using 3D echocardiography-based models: The updated ReVISION method. Front. Cardiovasc. Med..

[17] Szijarto A., Nicoara A., Podgoreanu M., Tokodi M., Fabian A., Merkely B., Sarkany A., Toser Z., Lakatos B.K., Kovacs A. (2024). Artificial intelligence-enabled reconstruction of the right ventricular pressure curve using the peak pressure value: A proof-of-concept study. Eur. Heart J.-Imaging Methods Pract..

[18] Lakatos B.K., Rako Z., Szijártó Á., da Rocha B.R.B., Richter M.J., Fábián A., Gall H., Ghofrani H.A., Kremer N., Seeger W. (2024). Right ventricular pressure-strain relationship-derived myocardial work reflects contractility: Validation with invasive pressure-volume analysis. J. Heart Lung Transplant..

[19] Naeije R., Richter M.J., Rubin L.J. (2022). The physiological basis of pulmonary arterial hypertension. Eur. Respir. J..

[20] Wang N., Rueter P., Ng M., Chandramohan S., Hibbert T., O’Sullivan J.F., Kaye D., Lal S. (2024). Echocardiographic predictors of cardiovascular outcome in heart failure with preserved ejection fraction. Eur. J. Heart Fail..

[21] Bay K., Gustafsson F., Maiborg M., Bagger-Bahnsen A., Strand A.M., Pilgaard T., Poulsen S.H. (2022). Suspicion, screening, and diagnosis of wild-type transthyretin amyloid cardiomyopathy: A systematic literature review. ESC Heart Fail..

[22] De Gregorio C., Trimarchi G., Faro D.C., Poleggi C., Teresi L., De Gaetano F., Zito C., Lofrumento F., Koniari I., Licordari R. (2024). Systemic Vascular Resistance and Myocardial Work Analysis in Hypertrophic Cardi-omyopathy and Transthyretin Cardiac Amyloidosis with Preserved Left Ventricular Ejection Fraction. J. Clin. Med..

[23] Urbano-Moral J.A., Gangadharamurthy D., Comenzo R.L., Pandian N.G., Patel A.R. (2015). Three-dimensional speckle tracking echocardiography in light chain cardiac amyloidosis: Examination of left and right ventricular myocardial mechanics parameters. Rev. Española Cardiol. (Engl. Ed.).

[24] Vitarelli A., Lai S., Petrucci M.T., Gaudio C., Capotosto L., Mangieri E., Ricci S., Germanò G., De Sio S., Truscelli G. (2018). Biventricular assessment of light-chain amyloidosis using 3D speckle tracking echocardiography: Differentiation from other forms of myocardial hypertrophy. Int. J. Cardiol..

[25] Mishra T., Pahuja M., Abidov A. (2019). Increasingly Recognized Role of Right Ventricle Assessment in Cardiac Amyloidosis. JACC Heart Fail..

[26] Uzan C., Lairez O., Raud-Raynier P., Garcia R., Degand B., Christiaens L.P., Rehman M.B. (2018). Right ventricular longitudinal strain: A tool for diagnosis and prognosis in light-chain amyloidosis. Amyloid.

[27] Licordari R., Minutoli F., Recupero A., Campisi M., Donato R., Mazzeo A., Dattilo G., Baldari S., Vita G., Zito C. (2021). Early impairment of right ventricular morphology and function in transthyretin-related cardiac amyloidosis. J. Cardiovasc. Echogr..

[28] Arvidsson S., Henein M.Y., Wikström G., Suhr O.B., Lindqvist P. (2018). Right ventricular involvement in transthyretin amyloidosis. Amyloid.

[29] Ghio S., Perlini S., Palladini G., Marsan N.A., Faggiano G., Vezzoli M., Klersy C., Campana C., Merlini G., Tavazzi L. (2007). Importance of the echocardiographic evaluation of right ventricular function in patients with AL amyloidosis. Eur. J. Heart Fail..

[30] Bodez D., Ternacle J., Guellich A., Galat A., Lim P., Radu C., Guendouz S., Bergoend E., Couetil J.-P., Hittinger L. (2016). Prognostic value of right ventricular systolic function in cardiac amyloidosis. Amyloid.

[31] Wan K., Lin J., Guo X., Song R., Wang J., Xu Y., Li W., Cheng W., Sun J., Zhang Q. (2020). Prognostic value of right ventricular dysfunction in patients with AL amyloidosis: Comparison of different techniques by cardiac magnetic resonance. J. Magn. Reson. Imaging.

[32] Garcia-Pavia P., Rapezzi C., Adler Y., Arad M., Basso C., Brucato A., Burazor I., Caforio A.L.P., Damy T., Eriksson U. (2021). Diagnosis and treatment of cardiac amyloidosis: A position statement of the ESC Working Group on Myocardial and Pericardial Diseases. Eur. Heart J..

[33] Bartolini S., Baldasseroni S., Fattirolli F., Silverii M.V., Piccioli L., Perfetto F., Marchionni N., Di Mario C., Martone R., Taborchi G. (2021). Poor right ventricular function is associated with impaired exercise capacity and ventilatory efficiency in transthyretin cardiac amyloid patients. Intern. Emerg. Med..

[34] Hashimoto H., Kurata A., Mizuno H., Nashiro T., Hangaishi A., Kuroda M., Usuki K., Horiuchi H. (2015). Pulmonary arterial hypertension due to pulmonary vascular amyloid deposition in a patient with multiple myeloma. Int. J. Clin. Exp. Pathol..

